# Carbon Transfer from the Host Diatom Enables Fast Growth and High Rate of N_2_ Fixation by Symbiotic Heterocystous Cyanobacteria

**DOI:** 10.3390/plants9020192

**Published:** 2020-02-04

**Authors:** Keisuke Inomura, Christopher L. Follett, Takako Masuda, Meri Eichner, Ondřej Prášil, Curtis Deutsch

**Affiliations:** 1 School of Oceanography, University of Washington, 1492 NE Boat St., Seattle, WA 98105, USA; cdeutsch@uw.edu; 2 Department of Earth, Atmospheric and Planetary Sciences, Massachusetts Institute of Technology, Cambridge, MA 02139, USA; follett@mit.edu; 3 School of Earth and Ocean Sciences, Cardiff University, Main Building, Park Place, Cardiff CF10 3AT, UK; 4 Institute of Microbiology, The Czech Academy of Sciences, 379 81b Třeboň, Czech Republic; takako@alga.cz (T.M.); eichner@alga.cz (M.E.); prasil@alga.cz (O.P.)

**Keywords:** DDA, nitrogen fixation, diatom, diazotroph, carbon, nitrogen, growth rate, photosynthesis, cell flux model

## Abstract

Diatom–diazotroph associations (DDAs) are symbioses where trichome-forming cyanobacteria support the host diatom with fixed nitrogen through dinitrogen (N_2_) fixation. It is inferred that the growth of the trichomes is also supported by the host, but the support mechanism has not been fully quantified. Here, we develop a coarse-grained, cellular model of the symbiosis between *Hemiaulus* and *Richelia* (one of the major DDAs), which shows that carbon (C) transfer from the diatom enables a faster growth and N_2_ fixation rate by the trichomes. The model predicts that the rate of N_2_ fixation is 5.5 times that of the hypothetical case without nitrogen (N) transfer to the host diatom. The model estimates that 25% of fixed C from the host diatom is transferred to the symbiotic trichomes to support the high rate of N_2_ fixation. In turn, 82% of N fixed by the trichomes ends up in the host. Modeled C fixation from the vegetative cells in the trichomes supports only one-third of their total C needs. Even if we ignore the C cost for N_2_ fixation and for N transfer to the host, the total C cost of the trichomes is higher than the C supply by their own photosynthesis. Having more trichomes in a single host diatom decreases the demand for N_2_ fixation per trichome and thus decreases their cost of C. However, even with five trichomes, which is about the highest observed for *Hemiaulus* and *Richelia* symbiosis, the model still predicts a significant C transfer from the diatom host. These results help quantitatively explain the observed high rates of growth and N_2_ fixation in symbiotic trichomes relative to other aquatic diazotrophs.

## 1. Introduction

Diatom–diazotroph associations (DDAs) are symbioses where the diazotrophs (e.g., *Richelia* and *Calothrix* sp.) are associated with diatoms (e.g., *Hemiaulus*, *Rhizosolenia*, and *Chaetoceros* sp.) [1,2,3,4,5,6,7]. They are widely observed [5,8,9,10,11,12,13,14,15,16] and predicted [17,18,19] in warm waters of the ocean. The symbiotic diazotrophs form a trichome where generally only one specialized cell, called a heterocyst, fixes dinitrogen (N_2_). The remaining cells in the trichome, called vegetative cells, are phototrophic and divide, whereas heterocysts do not. Despite the seemingly ideal combination of cells specialized for carbon (C) and nitrogen (N) acquisition, the trichomes have rarely been observed as free-living organisms in the marine environment [20,21]. This indicates that the trichomes receive some essential nutrients, which allow them to grow more efficiently as a part of the symbiosis. Recent studies revealed simplified N pathways in *Richelia* [7] and a significant amount of fixed N transferred to the diatom host from its symbiont [6]. The exchange of C between the diatom hosts and trichomes has been anticipated, but it has not been clearly demonstrated [2,22,23]. This is in contrast to cyanobacteria–plant symbiosis where the cyanobiont becomes photosynthetically inactive [23,24,25,26,27] and C transfer from the host has been directly observed [23,24,28,29,30].

In addition to the high rate of N_2_ fixation, a compilation of observed growth rate [31] shows a higher mean growth rate for DDAs than other, non-symbiotic, marine cyanobacterial diazotrophs. This enhanced growth is an essential assumption for an ecosystem model to reproduce observed seasonal blooms of DDAs in the oligotrophic ocean [31]. In general, the marine cyanobacterial diazotrophs grow at approximately 0.3 (d^−1^) under nutrient replete diazotrophic cultures [32,33,34,35,36], whereas *Richelia* in *Rhizosolenia*–*Richelia* symbiosis can grow as high as 0.87 (d^−1^) in diazotrophic conditions [1,2]. In addition, in situ studies show that the growth rate of *Crocosphaera* (unicellular diazotrophic cyanobacteria) is low (0.001–0.15 (d^−1^)) in comparison with *Richelia* in *Hemiaulus*–*Richelia* symbiosis, which grew up to 0.59 (d^−1^) [6]. What makes the high rates of N_2_ fixation and growth possible? Here, we seek to quantify the extent to which the enhanced growth and N_2_ fixation rates in the trichomes could be caused by the exchange of resources with the host diatom. 

**Figure 1:**
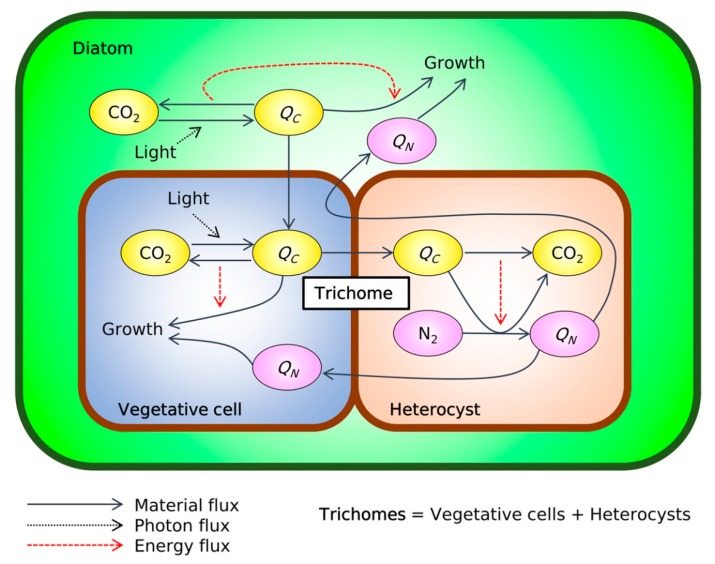
Schematic of the cell flux model of diatom–diazotroph association (CFM-DDA). Green frame: the silica frustule of the diatom (*Hemiaulus*). Brown frame: cell membrane layers of trichomes (*Richelia*). Green space: intracellular space of the diatoms. Blue space: intracellular space of vegetative cells. Orange space: intracellular space of heterocysts. Yellow ovals: C-based molecules. Pink ovals: N-based molecules. *Q_C_* and *Q_N_* indicate cellular quotas of C and N, respectively. A schematic with detailed notation is shown in Figure S1.

To quantitatively examine the host–trichome nutrient exchange, we have developed a coarse-grained model of the *Hemiaulus*–*Richelia* symbiosis (cell flux model of DDAs: CFM-DDA) adapting relevant parts from previous CFMs [37,38,39,40,41], such as an idealized metabolic-flux network constrained by mass, energy, and electron budget. Extensive quantitative characteristics exist for this symbiosis [6], including cell volume and the number of trichomes per diatom. The availability of these cellular characteristics and their relative consistency make this symbiosis an ideal candidate for modeling. The CFM-DDA model we develop here focuses on C and N metabolisms to quantify growth and N_2_ fixation (Figure 1). For most N_2_-fixing organisms, oxygen (O_2_) metabolism is important, since O_2_ damages the N_2_ fixing enzyme, nitrogenase, and may control the rate of N_2_ fixation [39,40,42,43,44]. However, since the trichomes form a heterocyst, a cell with a thick glycolipid layer to minimize O_2_ influx [45], we assume that intracellular O_2_ is managed with normal levels of respiration [37,46]. This simplification allows us to focus on the metabolisms of C and N as the basis of the symbiosis. O_2_ metabolism could straightforwardly be included in our modeling framework if required by future observations.

Here, we resolve only four processes (CFM-DDA, Figure 1): photosynthesis, biosynthesis, respiration, and N_2_ fixation. Photosynthesis and biosynthesis are done only by the host diatom and vegetative cells whereas N_2_ fixation is performed only by the heterocysts. Respiration is done by all cells and adjusts to meet the energetic demand for all the other processes. We scaled the rate of photosynthesis based on the cellular N quota, which was estimated from the typical cell volumes (3493.5 µm^3^ for a diatom, 18.8 µm^3^ for a vegetative cell, and 61.0 µm^3^ for a heterocyst) [6], empirical volume–C relationship [47], and an assumed elemental stoichiometry (C:N of 6.6) [48], as well as the ratio of vegetative–cell:heterocyst of 4:1 and two *Richelia* trichomes per diatom based on the available microscopic images [6,7,49,50] (see Methods). The predicted balance of photosynthesis and metabolic demand for C suggests that a significant amount of C is transferred from the host to the trichome, sustaining its high rate of N_2_ fixation and enhanced growth. 

## 2. Results and Discussion

### 2.1. Nitrogen Budget

**Figure 2:**
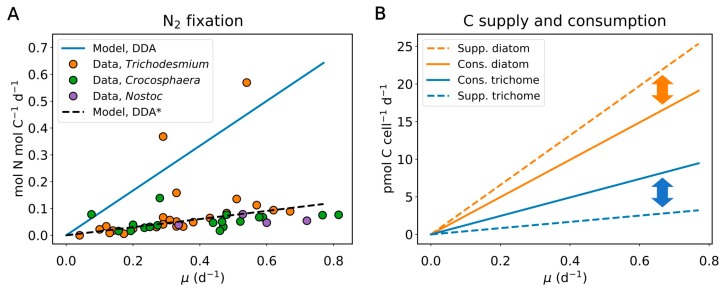
Rate of N_2_ fixation and the balance of C supply and consumption. (**A**) Rate of N_2_ fixation per C in the trichome. In the legend, “Model, DDA” indicates model results for DDA. “Model, DDA*” is model ignoring N_2_ fixation for diatom. “Data *Trichodesmium”*, “Data *Crocosphaera”,* and “Data *Nostoc”* are data for *Trichodesmium* [32,33,54], *Crocosphaera* [6,34,35,55,56], and *Nostoc* [57], respectively. (**B**) Predicted balance of C supply and consumption. Supp. and Cons. indicate “supply from” and “consumption by”, respectively. The arrows are to point out the supply–demand discrepancies.

**Figure 3:**
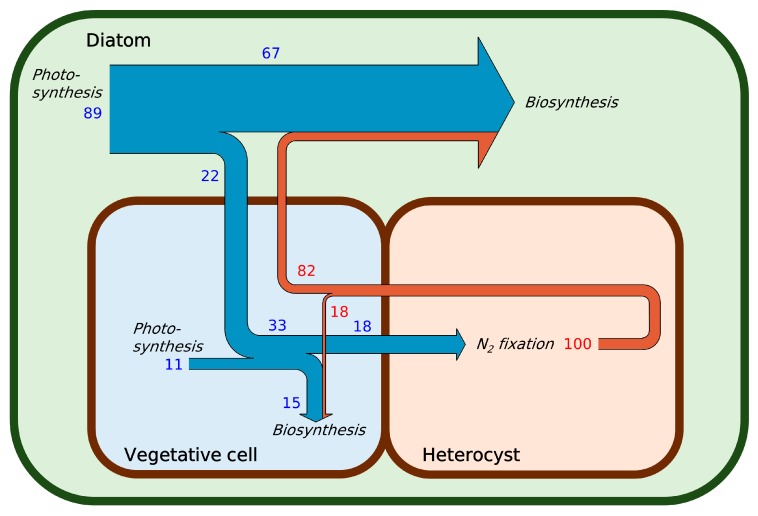
Simulated C (blue arrows) and N (brown arrows) exchanges between the diatoms (*Hemiaulus*) and trichomes (*Richelia*). The width of the arrows represents the relative magnitude of fluxes in mass. Blue numbers represent percentages of C fluxes (here, 100 indicates total sources and sinks of C in the symbiosis; e.g., 89 on the top left means 89% of photosynthesis in the symbiosis is done by a diatom) and red numbers are percentages of N (separate from those of C).

The model results suggest that the trichomes (*Richelia*) fix significantly more N per unit C than other photoautotrophic diazotrophs (Figure 2A). Based on the size differences, the diatom contains 4.5 times more N than the trichomes. Assuming that the diatom’s N demand is fully covered by the N_2_-fixing symbiont [1,6] and that both organisms grow at the same speed (which is required if the symbiosis is in steady-state growth [51,52]), the model predicts that the trichome must fix N_2_ 5.5 times faster than in the hypothetical case without the host. This increase has been observed in analogous systems. For example, an increase in N_2_ fixation has been reported for the *Anthoceros–Nostoc* symbioses as compared to free-living trichomes (4- and 35-fold increase relative to *Anabaena* and *Nostoc*, respectively [53]). Additionally, we find that the amount of N_2_ fixation done by the trichome to support its own growth falls in a similar range to that of other non-symbiotic aquatic N_2_-fixing organisms, including filamentous *Trichodesmium* [32,33,54], unicellular *Crocosphaera* [6,34,35,55,56], and a similar heterocyst-forming trichome from freshwater environments, *Nostoc* [57]. The similarity of N_2_ fixation rates suggests relatively conserved rates of N_2_ fixation across free-living diazotrophs. Moreover, the rate of N_2_ fixation by the DDA is significantly higher than these rates, indicating that the elevated rate of N_2_ fixation under symbiosis exists to fulfill the N demand of the diatom. Taken together, these results suggest that the rate of N_2_ fixation adjusts to meet the demand. The computed fate of N shows that 82% of fixed N flows into diatoms for their growth, whereas only 18% go to the new trichome (Figure 3). As the growth rate increases, the demand for fixed N increases, so the rate of N_2_ fixation must rise in proportion (Figure 2A).

### 2.2. Carbon Budget

Based on the photosynthetic capacity inferred from the cell volume, we predict that about 90% of photosynthesis by the *Hemiaulus*–*Richelia* symbiosis is done by *Hemiaulus* (the diatom) (Figure 2B and Figure 3). The largest part of fixed C (82%: 67% for diatom and 15% for the trichome (*Richelia*)) is used for biosynthesis (including respiration providing energy for biosynthesis), and the remaining 18% is used for supplying energy and electron for N_2_ fixation (Figure 3). A previous quantitative study on the heterotrophic soil N_2_ fixer *Azotobacter vinelandii* showed that the direct energy cost for N_2_ fixation is relatively small due to the overwhelming cost for managing O_2_ diffused from the environment [37]. However, since heterocysts most likely do not require such respiratory protection due to the thick glycolipid layer [37], the direct cost for N_2_ fixation (energy and electron for N_2_ reduction) is quantitatively significant in the symbiosis. The model predicts that about 10% of the total photosynthesis is achieved by vegetative cells in the symbiosis (Figure 2B and Figure 3). This is higher than that in the *Azolla*–*Anabaena* symbiosis where *Anabaena* accounts for only less than 5% of photosynthesis [23,28,30]. However, even if the *Richelia* trichomes use all the C fixed by themselves for N_2_ fixation, there is still a deficit in C, and the trichome requires even more C to support its own biosynthesis.

With these factors, the model predicts the imbalances between the supply and demand of C for the trichome and for the diatom (Figure 2B and Figure 3). Due to the high rate of N_2_ fixation and its relatively high cost together with the cost for the biosynthesis, one-third of the total fixed C is consumed in the trichomes, which is about three times the C supplied by their own photosynthesis (Figure 3). On the other hand, since diatoms do not directly pay the cost for N_2_ fixation, the C they generate exceeds their own requirements if the C demand and supply are balanced (Figure 3). These imbalances in the C budget between the different organisms indicate that there is a C flux from the diatom to the trichome. The model predicts that about one-fourth of the C fixed by diatoms flows into the trichome, supporting their N_2_ fixation rates (Figure 3). 

**Figure 4:**
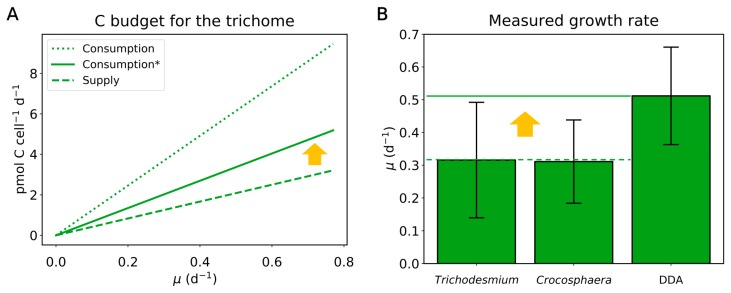
Simulated C budget for the *Richelia* trichomes and measured growth rates. (**A**) Cost and supply of C by the trichome per diatom (*Hemiaulus*) cells. Consumption* indicates the consumption of C that ignores the C consumption for N_2_ fixation for diatoms. (**B**) Measured growth rates of marine free-living diazotrophic cyanobacteria and DDAs (compiled by Follett et al., 2018) [31].

The predicted amount of C transfer supports more than just N_2_ fixation for the diatom’s needs. Even if we ignore the cost of N_2_ fixation for the diatom, the total modeled C cost for the trichome is still greater than its photosynthesis provides. About 60% more C is consumed than they produce, with the remainder coming from the host diatom (Figure 4A). This is based on that the diatom does not allocate its own space for N_2_ fixation whereas the trichome allocates a significant space for the heterocyst (approximately 43% in C in the trichome) to N_2_ fixation at the exclusion of photosynthesis. This additional C supply from the diatom may contribute to the observed faster growth rate of DDAs compared to non-symbiotic diazotrophs (Figure 4B). Given nutrient-replete conditions (except for N, all of which is supplied by N_2_ fixation), the growth rate of the trichome, *μ_Trichome_*, would be limited by the C supply rate and the yield (the efficiency of C use). Since the yield is fixed, the *μ_Trichome_* is proportional to the C supply rate [39]. C supply from the diatom increases the total C supply in the trichome by about 60% (Figure 4A), which increases *μ_Trichome_* by the same proportion as well. This increase in *μ_Trichome_* resembles the difference between the measured growth rates of non-symbiotic diazotrophs and DDAs (Figure 4B), indicating that the C transfer could be a major factor enabling the faster growth of DDAs. 

### 2.3. The Influence of the Diatom Size, Trichome Counts, and Light Harvesting by Heterocysts

**Figure 5:**
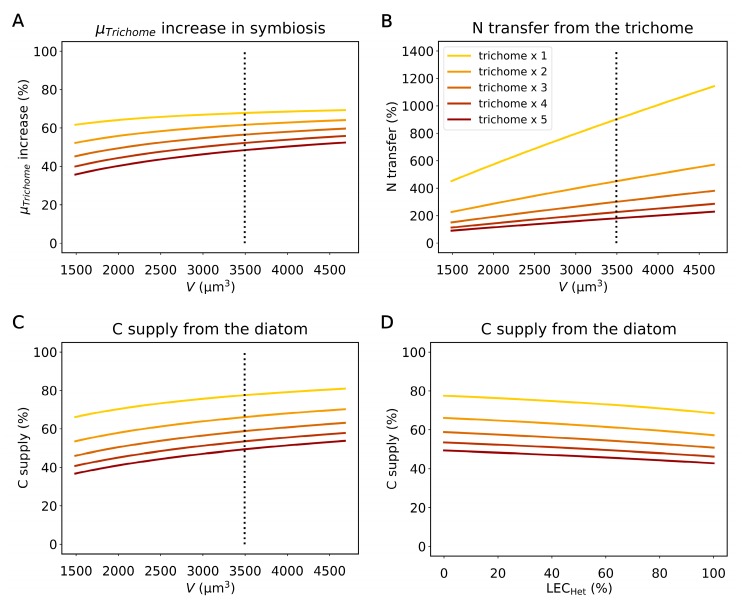
Simulated influences of the diatom volume (*V*) and number of the trichomes per diatom on N and C exchanges and the impact of light energy supply in heterocysts for sensitivity tests. (**A**) Increase in the (nutrient replete) growth rate of the trichomes (*μ_Trichome_*) relative to that for the trichome without symbiosis. (**B**) N transfer from the trichome to diatom relative to the N requirement for the trichome for various diatom sizes and number of the trichomes; 100% means that the amount of N transfer equals the requirement for the growth of the trichomes. (**C**) C supply from the diatom relative to the total C requirement of the trichome. Dotted lines indicate the averaged volume based on the observations after removing the two highest outliers [6]. (**D**) The impact of light energy contribution in heterocysts (LEC_Het_) on C supply from the diatom relative to the total C requirement of the trichome. LEC_Het_ indicates the fraction of energy requirements for N_2_ fixation that is covered by light harvesting in heterocysts. For example, 100% means no fixed C requirement for energy production for N_2_ fixation. We note that this energy requirement differs from the electron requirement for N_2_ fixation, which may not be covered by the light harvesting due to a lack of active Photosystem II [58]. The legend in (B) applies to all the panels. Trichome × *n* indicates *n* trichome per a diatom cell.

In the above simulations, we used the “average” cell sizes and number of the *Richelia* trichomes per diatom (*Hemiaulus*) cell to quantify a typical map of material exchange (Figure 3; see Methods). However, in nature, these numbers vary [6]. Here, we simulated various possible cell sizes of diatoms (1490–4680 µm^3^) [6] and numbers of the trichomes per diatom (1–5) to quantify how they influence N and C transfer (Figure 5A–C). The trichome growth rate is sensitive to these factors, since they change the diatom–trichome volume ratio (Figure 5A). As the diatoms size increases, a relatively higher amount of C is fixed by the diatom, and more C can be used for the growth of the trichome, resulting in increased *μ_Trichome_*. Similarly, as the number of the trichomes per diatom increases, the photosynthesis of the diatom per trichome decreases, and the *μ_Trichome_* decreases.

N transfer from the trichome is also sensitive to the volume of the diatom. Increasing the diatom size demands more N_2_ fixation (Figure 5B), as it leads to increased cellular quotas of N. N transfer from the trichome increases linearly, reflecting the linear relation between the volume and cellular N quota. In the default run with two trichomes per diatom with the averaged size (3493.5 µm^3^), about 450% of fixed N (relative to the need for the trichomes) flows into the diatom, which increases to 901% when there is only one trichome. It is notable that even with five trichomes, which are hardly observed for *Hemiaulus–Richelia* symbiosis [6,7,49,50], 180% of fixed N is transferred to the host diatom.

The C supply from the diatom reflects the cost for excess N_2_ fixation. As the diatom volume increases, the cost for N_2_ fixation increases (Figure 5B), which in turn raises the C demand and thus the transfer of C from the diatom (Figure 5C). In the default run with the average diatom (*Hemiaulus*) size and 2 trichomes, 66% of C for the trichomes’ metabolic demand depends on the diatom, which increases to 78% when there is only one trichome (Figure 5C). When there are five trichomes, the cost decreases, but there is still about 50% of C transferred from the host diatom, supporting the idea that the C transfer from the diatom is essential, which may explain why *Richelia* is an obligate symbiont [7]. We have also tested how many vegetative cells are needed to make the trichome independent from the C supply from the host by using the obtained photosynthesis rate per vegetative cell and varying the number of vegetative cells per heterocyst. The result shows that when there are two trichomes (as in the default run), 104 vegetative cells would be required for supporting the growth of trichomes and N_2_ fixation for the symbiosis without C transfer from the host (Figure S2). If this was the case, the total volume of the trichomes would be larger than the diatom (Figure S2), supporting that C transfer is necessary.

Heterocysts do not have active Photosystem II (PSII) but possess Photosystem I (PSI) [58,59]. Thus, although they cannot generate reducing equivalents (electrons) by linear photosynthetic electron transport, they can potentially harness light energy and generate ATP for N_2_ fixation using PSI [58]. We have simulated various light energy contributions to N_2_ fixation within heterocysts (LEC_Het_) for another sensitivity test (Figure 5D). When all the ATP for N_2_ fixation is covered by light harvesting in the heterocyst (thus, LEC_Het_ = 100%), it reduces the relative C supply from the diatom by less than 10%. The absolute value of reduction is especially significant when there is only one trichome in a diatom, since the per trichome rate of N_2_ fixation is the highest. However, even when 100% of the energy (ATP) requirement for N_2_ fixation is covered by the light harvesting in the heterocyst, we still predict a significant C supply from the diatom because of the large C requirement for providing electrons to reduce N_2_ (one C per N [37,38,39]). The model predicts that this C cost is similar to that for providing ATP for N_2_ fixation; thus, about half of the N_2_ fixation cost must be still paid even after all the ATP requirement is waivered.

### 2.4. Implication of the Model Results for Other DDAs 

In this study, we have developed a cell flux model of DDAs (CFM-DDA) based on *Hemiaulus*–*Richelia* symbiosis and predicted C and N exchanges. How does the model results apply to other DDAs such as *Rhizosolenia*–*Richelia* [2,22,50] and other related symbioses, such as *Chaetoceros*–*Calothrix* (or *Richelia*) [7,9]? The framework of CFM-DDA should apply to these other symbioses since the modeled coarse-grained metabolisms are general enough to capture the common metabolism of the diatoms and trichomes and potential nutrient exchanges. In addition, the model prediction of C supply from the host to the trichome resonates with plant–cyanobacterial symbiosis where C transfer from the plant to the cyanobacteria has been observed [23,24,28,29,30]. *Richelia* is an obligate cyanobiont [7], indicating that it receives essential molecules from the host diatom including *Rhizosolenia*. Furthermore, our prediction of C transport is qualitatively supported by the observation where the *Calothrix* trichomes increase the number of the vegetative cells once they are detached from the host [60], which is likely to compensate for a lack of C supply from the diatom. 

However, despite the generality of the model framework and these supporting implications, there are fundamental differences between the different DDAs, requiring any extrapolation of these results to be used with caution. For example, *Rhizosolenia* has a large vacuole [61], where photosynthesis does not occur, potentially leading to overestimation of the photosynthesis. In addition, *Richelia* in *Rhizosolenia* have higher numbers of vegetative cells per heterocyst than that in *Hemiaulus* [2,62,63], indicating higher C supply from the trichome. The number of the trichomes per host cell can be higher for *Rhizosolenia* as well [2,50,64]. Furthermore, the trichomes are often externally associated with *Chaetoceros* [2,9,31]; thus, the exchange of molecules may be less efficient. For example, if they are transported through the external environment, a significant loss must be associated.

To address some of the factors, we have extended our sensitivity study with an increased number of vegetative cells (10 per heterocyst), more trichomes per diatom (up to 15 per diatom), and a wider range of cellular volume (1000–7000 µm^3^: lower cell volume would have equivalent effects as higher vacuole amount) (Figure S3). Although these factors decrease the amount of C transported, we still predict supply–demand imbalance in C, thus transferring the C from the host (>10% of C supply from the host even in extreme scenarios). In addition, if there is a loss of N to the environment, it would increase the C demand, thus resulting in higher C supply from the host diatom. To test our predictions, further experiments would be required as described at the end of the next section.

### 2.5. Hypothesis: Mechanism for High C Processing

High rates of N_2_ fixation by DDAs have been observed, but the mechanisms for that have not been fully elucidated. With a simple but mechanistic model for DDAs, we predict that a significant C flux from the diatom enhances both the growth and N_2_ fixation rates within the trichome. This leads to a further question: What mechanisms may allow the cell to process such a high amount of C at a faster rate than non-symbiotic diazotrophs? Here, we consider this question based on the protein allocation within the cell and suggest that parts of the biosynthesis pathway might occur within the diatom rather than the trichome.

Proteins (enzymes) are responsible for most of the biochemical reactions, and increasing the growth rate requires a higher density of growth-related proteins [65,66,67,68]. However, cellular space (resource) is limited, and cells need to allocate the finite amount of proteins for various purposes [65,66,69,70]. A coarse-grained model parameterized using laboratory measurements show that increasing the allocation of proteins related to amino acid synthesis sacrifices growth, because a smaller amount of proteins is allocated to growth-related proteins [66]. The reverse must also be true: If cells allocate a smaller amount of proteins for amino acid synthesis, more proteins can be allocated to the growth-related proteins, which should lead to faster growth.

If this theory is applied to the symbiosis, where molecular exchanges are possible between organisms, it is possible that one organism may effectively increase the growth rate of the other while sacrificing its own. This could be accomplished with altered protein allocation. For example, in DDAs, if most amino acid synthesis is done by diatoms, the diatom’s growth rate would decrease, since a higher amount of proteins must be allocated for amino acid synthesis. However, this would increase the growth of the trichomes, since they can reduce their allocation toward amino acid synthesis in favor of growth-related proteins. In the context of protein allocation, the reduced protein allocation for amino acid synthesis can also lead to a higher allocation of the N_2_-fixing enzyme: nitrogenase.

**Figure 6:**
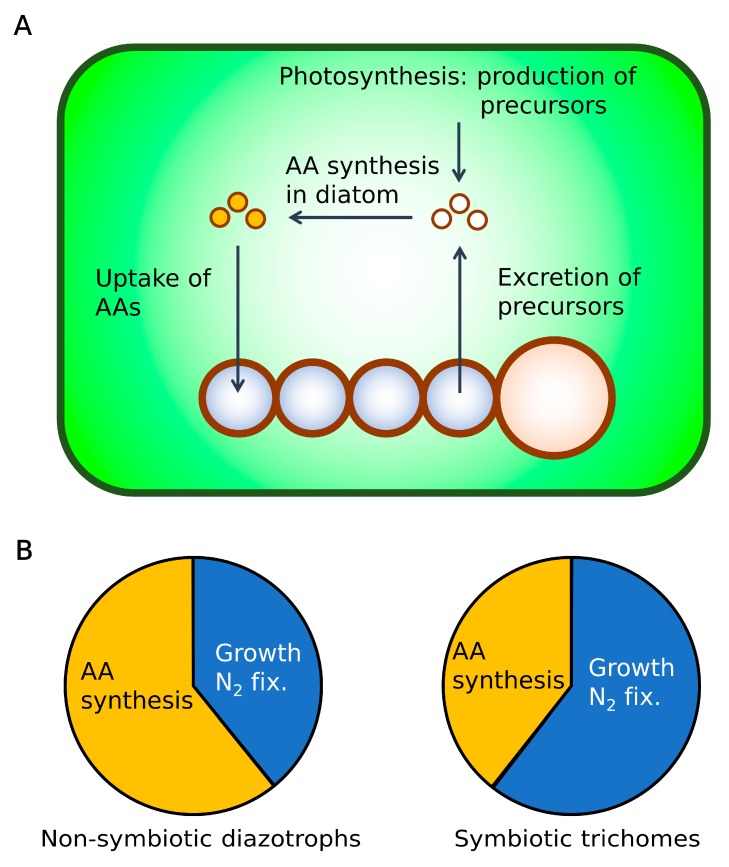
Hypothetical view of molecular exchanges between the diatom and the trichome and the protein allocation of the trichome. (**A**) Molecular exchanges between diatom and the trichome. Green space: cytoplasm in the diatom. Blue circles: vegetative cells. Orange Space: heterocysts. (**B**) The idealized protein allocations of non-symbiotic diazotrophs and symbiotic trichomes. Here, only proteins (enzymes) for amino acid (AA) synthesis and growth and N_2_ fixations (N_2_ fix.) are considered for conceptual simplicity. By having the diatom process molecules, symbiotic trichomes would be able to allocate more proteins for growth and N_2_ fixation, allowing the cells to grow and fix N_2_ faster.

Thus, we hypothesize that diatoms “help” synthesize amino acids for the trichomes, leading to a reduced allocation of proteins for amino acid synthesis but an increased allocation toward growth-related proteins and nitrogenase in the trichomes. This leads to higher growth rates and faster C processing within the trichomes (Figure 6). This hypothesis is supported by various observations. First, the growth rate of diatoms in DDAs is lower than that of their free-living counterparts [31], indicating that they experience additional metabolic costs within the symbiosis. In addition, the *Richelia* trichome maintains amino acid transporters, despite the loss of various other transporters, enabling the uptake of amino acids from the diatom [7]. Additionally, the trichomes do not grow [7] or grow only at a slower pace once they are detached from the host [6], suggesting a reliance on diatoms for some molecules, possibly specific amino acids. Furthermore, *Richelia* in *Hemiaulus* and *Calothrix* in *Chaetoceros* have a high ratio of heterocysts (1 out of approximately 5 cells [2,6,7,49,50,71]), whereas free-living *Anabaena* in average have approximately 10 vegetative cells between heterocysts under diazotrophic conditions [72,73,74,75,76,77,78], supporting the increased nitrogenase content in the trichomes in these specific DDAs. 

Our hypothesis can be tested with the combination of different levels of omics, imaging techniques, and kinetic analysis on field-sampled DDAs and cultures of the symbionts. Genomics and transcriptomics obtained with and without a host would allow us to target the “product”, which is transported from diatoms and should be confirmed by proteomics and metabolomics. A combination of genomics, proteomics, and metabolomics will give information on the metabolic capacity, activity, and metabolic relationships between the diatom and the trichome. Following up on the previous NanoSIMS study of ^15^N fixation and transfer in DDAs [6], time course analyses and pulse chase experiments with ^13^C using NanoSIMS and Raman microscopy would allow us to compare C fixation rates between trichomes and diatoms, confirm the transport of biomolecules from diatoms to trichomes, and identify transferred biomolecules [79,80,81,82]. Analyses of C isotope fractionation in the diatom and trichome biomass should indicate in which of the cells the C was assimilated [83,84]. These results should be confirmed by rate measurements of growth, DNA synthesis, and protein synthesis. 

## 3. Conclusions

DDAs are major N_2_ fixers in the ocean whose rate of N_2_ fixation is quantitatively significant [6], but the connection between their metabolic rates and symbiotic association are unknown. With a simple mechanistic model of cellular metabolisms of *Hemiaulus*–*Richelia* symbiosis, we predict that the observed high rates of N_2_ fixation and growth of the trichomes [6,31] are supported by the C transfer from the host diatom, which is qualitatively consistent with the observations of plant–cyanobacteria symbiosis [23,24,28,29,30]. Our model also explicitly accounts for the C cost for N_2_ fixation, which is a central factor in the competitive fitness of diazotrophs relative to other plankton. The growth rate handicap by DDAs is commonly expressed as a constant factor in ecological and biogeochemical models [17,18,19], whereas our model shows that it is dependent on the molecular exchanges. Similarly, the model enables various cell sizes and number of the trichome per diatom, as well as the ratio of vegetative cells to the heterocysts, allowing the material exchanges and their metabolic advantages to be computed from mechanistic considerations. Such model flexibility allows the expression of diverse DDAs and can be used to study how such diversity helps DDAs to acquire their roles as significant sources of bioavailable N.

## 4. Methods

The CFM-DDA (Figure 1) is based on the following core equation, a steady-state solution for the time dependences of each C and N pool (see Supplementary Methods and Figure S1 for the derivation):

(1) $$F_{Pho}^{D}+F_{Pho}^{V}=\mu \left(Q_{C}^{V}+Q_{C}^{H}+Q_{C}^{D}\right)\left(1+E\right)+\mu \left(Q_{N}^{V}+Q_{N}^{H}+Q_{N}^{D}\right)Y_{C:N}^{N2fix}$$

where $F_{Pho}^{D}$ and $F_{Pho}^{V}$ (pmol C d^−1^ cell^−1^) are the daily rate of per-DDA photosynthesis by diatoms (*Dia*) and vegetative cells (*Veg*), respectively, *μ* is the growth rate (d^−1^), $Q_{C}^{V}$, $Q_{C}^{H}$, and $Q_{C}^{D}$ are the cellular C quotas of *Veg*, *Het* (heterocysts), and *Dia* per DDA, $Q_{N}^{V}$, $Q_{N}^{H}$, and $Q_{N}^{D}$ are their respective N quotas per DDA, *E* is the ratio of respiration to biosynthesis, and $Y_{C:N}^{N2fix}$ is a conversion term from N_2_ fixation to its C cost (mol C mol N^−1^).

This equation represents the balance between the C supply (left-hand side) and C consumption (right-hand side). The terms on the left are the photosynthesis from each organism providing C to the system. The first term on the right-hand side represents the consumption of C based on biosynthesis (making new cells) and respiration for supporting it. The second term represents the C cost based on N_2_ fixation. 

We scale the rates and quotas based on the cell volume (*V*) (µm^3^). To convert *V* to C quotas, we have used a power relationship based on the compilation of various phytoplankton species [47]. This study suggests different relationships for non-diatom phytoplankton and diatoms: (pg C cell^−1^) = 0.216 × *V*^0.939^ and (pg C cell^−1^) = 0.288 × *V*^0.811^, respectively. Thus, we used the former equation for $Q_{C}^{V}$ and $Q_{C}^{H}$ and the latter for $Q_{C}^{D}$. We convert these C quotas to N quotas ($Q_{N}^{V}$, $Q_{N}^{H}$, and $Q_{N}^{D}$) based on the Redfield ratio of 6.6 C:1 N, following previous studies [6,48]. *E* is obtained based on the energy balance between biosynthesis and respiration with an energy transfer efficiency of 0.6 [85]. $Y_{C:N}^{N2fix}$ is based on the sum of the C costs for providing electron and energy to N_2_ fixation [37,38] with the same energy transfer efficiency as that for biosynthesis (0.6) [85] (see details in Supplementary Methods). As we have now defined all the values for the left-hand side, we can obtain ($F_{Pho}^{D}+F_{Pho}^{V}$) as a solution of Equation (1). To partition $F_{Pho}^{D}$ and $F_{Pho}^{V}$, we assume that the rates of photosynthesis are proportional to the cellular N quotas, since the size of the N quota indicates the enzyme availability for photosynthesis. We have used the averaged cell volume for each cell from observations of *Hemiaulus* and *Richelia* [6] ignoring two extraordinary large symbioses and a ratio of diatom:trichome of 1:2 to represent commonly observed relations between *Hemiaulus* and *Richelia* based on microscopic images [6,7,49,50] except for Figure 5, where the cell volume and number of the trichomes are varied: 1490–4680 (µm^3^) (a range from the observation [6] after ignoring the two large outliers) and 1–5 (between minimum and significantly higher value than generally observed), respectively. We used a ratio of *Veg*:*Het* of 4:1 to represent typically observed *Richelia* trichomes in *Hemiaulus* based on microscopic images [6,7,49,50]. 

## Supplementary Materials

The following are available online at https://www.mdpi.com/2223-7747/9/2/192/s1. Supplementary Methods. Figure S1: Schematic of CFM-DDA with flux notations. Figure S2: The impact of the number of vegetative cells per heterocyst on the C supply and demand and space occupation by the trichomes in a diatom cell. Figure S3: Sensitivity test for C supply from the diatom relative to the total C requirement of the trichome.

## Model Availability

CFM-DDA is freely available from Zenodo at https://doi.org/10.5281/zenodo.3561255 (DOI: 10.5281/zenodo.3561255).
