# Supplementary material for: Carbon Transfer from the Host Diatom Enables Fast Growth and High Rate of N2 Fixation by Symbiotic Heterocystous Cyanobacteria
Source: Plants (Basel). 2020 Feb 4;9(2):192. doi: 10.3390/plants9020192 (PMC7076409; doi:10.3390/plants9020192)
Supplement: Supplementary file 1 [file plants-09-00192-s001.pdf]

Supplementary material:

Supplementary Methods

Derivation of the key equation

Here we describe the derivation of Equation (1) in the main text. Figure S1 shows a schematic of CFM-DDA with flux notations. Below Q_i^j (pmol cell^{-1}) indicates the cellular quota of *i* in *j*, and J_i^{jk} (pmol cell^{-1} d^{-1}) indicates transport of *i* from *j* to *k*, where *i* = C or N and *j*,*k* = *H* (Heterocysts: *Het*), *V* (Vegetative cells: *Veg*) or *D* (Diatoms: *Dia*). We start with describing time dependences of each element in each cell. The balance of Q_C^H is based on C transfer and cost of N_2 fixation F_C^{N2fix} (pmol N cell^{-1} d^{-1}), which includes C cost for providing electron and energy through respiration:

$$\frac{dQ_C^H}{dt} = J_C^{VH} - F_C^{N2fix} \quad \text{[Eq. S1]}$$

Similarly, the time dependence of Q_N^H is based on the balance between fixed N transport and N_2 fixation F_N^{N2fix} (pmol N cell^{-1} d^{-1}):

$$\frac{dQ_N^H}{dt} = -J_N^{HV} + F_N^{N2fix} \quad \text{[Eq. S2]}$$

Veg needs to produce biomass both for new *Veg* and *Het*. Also, they can fix C. Thus, the time variation of C in *Veg* is as follows:

$$\frac{dQ_C^V}{dt} = F_{Pho}^V + J_C^{DV} - J_C^{VH} - \mu(Q_C^V + Q_C^H)(1 + E) \quad \text{[Eq. S3]}$$

where F_{Pho}^V (pmol C cell^{-1} d^{-1}) is photosynthesis (C fixation) by *Veg*, μ (d^{-1}) is growth rate and *E* (dimensionless) is respiration factor. This equation also includes the transfer of C from *Dia* and transfer of C to *Het*. Regarding N, however, *Veg* needs to acquire N from *Het* for biomass production for both new *Veg* and *Het* and for transporting N to *Dia*:

$$\frac{dQ_N^V}{dt} = J_N^{HV} - J_N^{VD} - \mu(Q_N^V + Q_N^H) \quad \text{[Eq. S4]}$$

The balance of C in *Dia* is similar to that in *Het* but its biomass production is only for themselves and transfer is only to support other cells:

$$\frac{dQ_C^D}{dt} = F_{Pho}^D - J_C^{DV} - \mu Q_C^D(1+E) \qquad \text{[Eq. S5]}$$

The balance of N is much simpler:

$$\frac{dQ_N^D}{dt} = J_N^{VD} - \mu Q_N^D \qquad \text{[Eq. S6]}$$

i.e., the balance between N supply from the heterocysts and the consumption for new-cell synthesis.

Once we get all the above balances, we assume the steady state where the time variation terms (i.e. dy/dt) go zero. Then we can eliminate transfer terms J_i^{jk}. By doing so, from [eq. S1, S3 and S5] we obtain

$$F_{Pho}^D + F_{Pho}^V = \mu(Q_C^V + Q_C^H + Q_C^D)(1+E) + F_C^{N2fix} \qquad \text{[Eq. S7]}$$

This equation is a simple representation of C balance for the entire symbiosis. The left-hand side represents the supply from the photosynthesis and the right-hand side is the consumption for biomass production, associate respiration and N_2 fixation. In a similar way, we can obtain the whole system balance in N from [eq. S2, S4 and S6]:

$$F_N^{N2fix} = \mu(Q_N^V + Q_N^H + Q_N^D) \qquad \text{[Eq. S8]}$$

The term on the left is the supply of N by N_2 fixation and the term on the right is the N consumption for biomass production. C consumption and fixed N production by N_2 fixation can be stoichiometrically related:

$$F_C^{N2fix} = F_N^{N2fix} Y_{C:N}^{N2fix} \qquad \text{[Eq. S9]}$$

where $Y_{C:N}^{N2fix}$ (mol C mol N^{-1}) is the ratio of C consumption and N fixation as represented as the sum of two terms:

$$Y_{C:N}^{N2fix} = Y_{C:N}^{N2fix,El} + Y_{C:N}^{N2fix,En} \qquad \text{[Eq. S10]}$$

where $Y_{C:N}^{N2fix,El}$ (mol C mol N^{-1}) is the fixed C consumption for electron donation for reducing N_2 per fixed N, and the second term $Y_{C:N}^{N2fix,En}$ is the fixed C consumption for energy production for N_2 fixation per fixed N. Values for these terms are obtained based on the previously developed method in CFM [1,2] with the energy transfer efficiency of 0.6 [3]. With [Eq. S9] through N_2 fixation terms [Eq. S7] and [Eq. S8] are combined:

$$F_{Pho}^{D} + F_{Pho}^{V} = \mu(Q_C^V + Q_C^H + Q_C^D)(1 + E) + \mu(Q_N^V + Q_N^H + Q_N^D)Y_{C:N}^{N2fix} \quad \text{[Eq. S11]}$$

and the key equation in this study (Equation 1) is now obtained.

Unit conversion of the data

In Figure 2A, we have compiled the data for N_2 fixation rates (mol N (mol C)$^{-1}$ d^{-1}). Since the data we referred to originally had different units, we had to convert them to be comparable with the model output. For converting the weight of chlorophyll to moles, we used 893.5 g Chl mol^{-1}. For converting chlorophyll to C in *Trichodesmium* and *Nostoc*, since the appropriate data for *Nostoc* could not be found, we used 208.3 μmol Chl (mol C)$^{-1}$, an intermediate value from compiled data on *Trichodesmium* [4]. We also converted biomass-specific rates of N_2 fixation (d^{-1}) to (mol N (mol C)$^{-1}$ d^{-1}) by dividing them by measured C:N ratios (mol C (mol N)$^{-1}$) [5]. Some N_2 fixation rates for *Crocosphaera* are given per cell. To normalize them to C, we used 184.8 fmol C cell^{-1}, an averaged value from two studies [6,7]. Also, to convert biomass-specific rates of N_2 fixation by *Crocosphaera*, we use an averaged C:N ratio (7.06 mol C (mol N)$^{-1}$) from these studies, using the middle value in cases where the data are presented as a range. To convert (h^{-1}) to (d^{-1}) we multiplied by 12 since the compiled organisms fix N_2 almost exclusively during either the light period or the dark period [8,9].

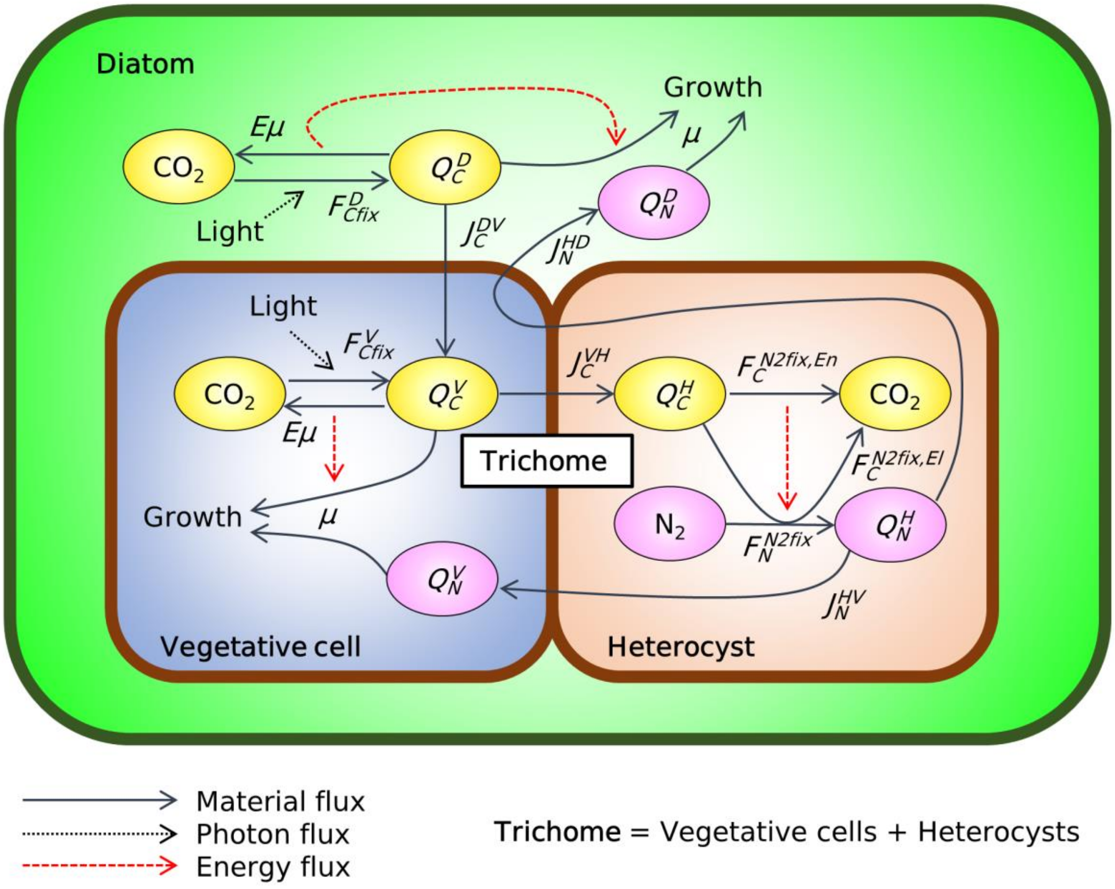

Figure S1 Schematic of CFM-DDA with flux notations. Green frame: the silica frustule of the diatom. Brown frame: cell membrane layers of the trichome. Green space: intracellular space of Diatoms. Blue space: intracellular space of vegetative cells. Orange space: intracellular space of heterocysts. Yellow ovals: C based molecules. Pink ovals: N based molecules. See Supplementary text for flux and quota notations. See Supplementary Methods for flux notations. $E\mu$ represents $E \times \mu$, growth-dependent respiration.

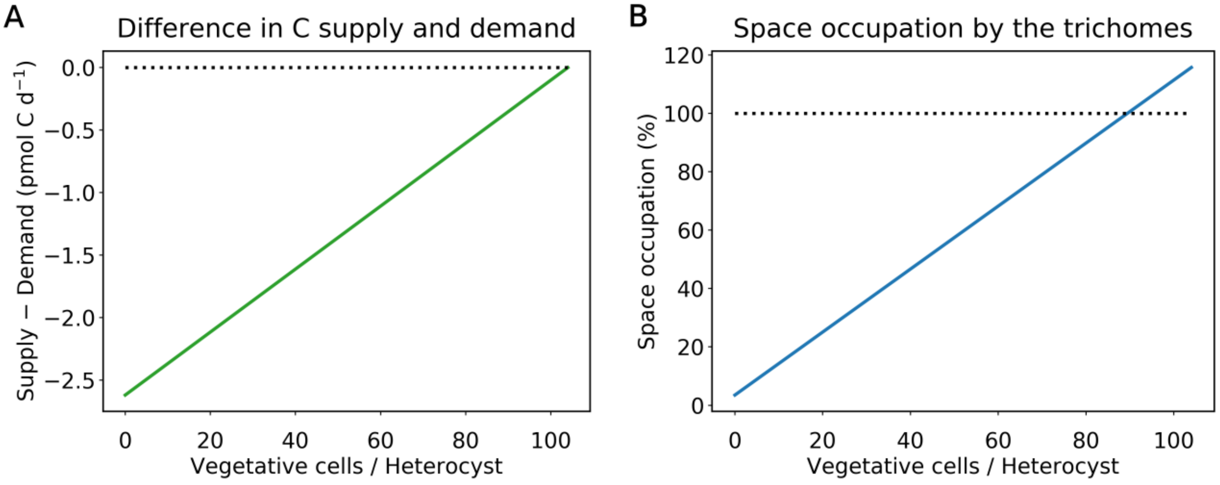

Figure S2 The impact of the number of vegetative cells per heterocyst on the C supply and demand and space occupation by the trichomes in a diatom cell. (**A**) Difference in C supply and demand of trichomes (supply – demand) assuming no C transfer from the diatom. (**B**) The fraction of intracellular space of a diatom occupied by the trichomes (volume of two trichomes / volume of one diatom × 100%).

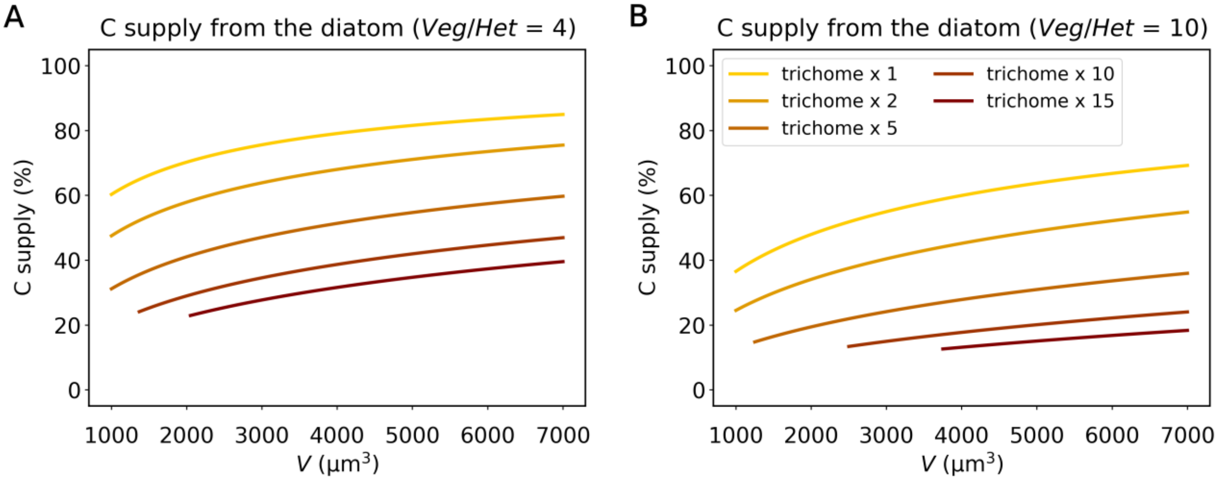

Figure S3 Sensitivity test for C Supply from the diatom relative to the total C requirement of the trichome. The number of vegetative cells per heterocyst (*Veg*/*Het*) is (**A**) 4 and (**B**) 10. Different colors indicate different number of trichomes per diatom (see legend in B). *V* in *x*-axis is volume. Results are not plotted where the volume of the diatom is samller than the total volume of the trichomes.

References

1. Inomura, K.; Bragg, J.; Follows, M.J. A quantitative analysis of the direct and indirect costs of nitrogen fixation: a model based on *Azotobacter vinelandii*. *ISME J.* **2017**, *11*, 166–175.

2. Inomura, K.; Bragg, J.; Riemann, L.; Follows, M.J. A quantitative model of nitrogen fixation in the presence of ammonium. *PLoS ONE* **2018**, *13*, e0208282.

3. Rittmann, B.E.; McCarty, P.L. Environmental Biotechnology: Principles and Applications. McGraw-Hill: New York, NY, USA. **2001**, 126-164.

4. LaRoche, J.; Breitbarth, E. Importance of the diazotrophs as a source of new nitrogen in the ocean. *J. Sea Res.* **2005**, *53*, 67–91.

5. Holl, C.M.; Montoya, J.P. Diazotrophic growth of the marine cyanobacterium *Trichodesmium* IMS101 in continuous culture: Effects of growth rate on N_2-fixation rate, biomass, and C:N:P stoichiometry. *J. Phycol.* **2008**, *44*, 929–937.

6. Großkopf, T.; LaRoche, J. Direct and indirect costs of dinitrogen fixation in *Crocosphaera watsonii* WH8501 and possible implications for the nitrogen cycle. *Front. Microbiol.* **2012**, *3*, 236.

7. Dron, A.; Rabouille, S.; Claquin, P.; Le Roy, B.; Talec, A.; Sciandra, A. Light-dark (12:12) cycle of carbon and nitrogen metabolism in *Crocosphaera watsonii* WH8501: relation to the cell cycle. *Environ. Microbiol.* **2012**, *14*, 967–981.

8. Berman-Frank, I.; Lundgren, P.; Falkowski, P. Nitrogen fixation and photosynthetic oxygen evolution in cyanobacteria. *Res. Microbiol.* **2003**, *154*, 157–164.

9. Mohr, W.; Intermaggio, M.P.; LaRoche, J. Diel rhythm of nitrogen and carbon metabolism in the unicellular, diazotrophic cyanobacterium *Crocosphaera watsonii* WH8501. *Environ. Microbiol.* **2010**, *12*, 412–421.
